# An Improved Genome-Scale Metabolic Model of *Arthrospira platensis* C1 (*i*AK888) and Its Application in Glycogen Overproduction

**DOI:** 10.3390/metabo8040084

**Published:** 2018-11-26

**Authors:** Amornpan Klanchui, Sudarat Dulsawat, Kullapat Chaloemngam, Supapon Cheevadhanarak, Peerada Prommeenate, Asawin Meechai

**Affiliations:** 1 Biological Engineering Program, Faculty of Engineering, King Mongkut’s University of Technology Thonburi, Bangkok 10140, Thailand; oofarto3@gmail.com; 2 Pilot Plant Development and Training Institute, King Mongkut’s University of Technology Thonburi (Bang Khun Thian), Bangkok 10150, Thailand; sudarat.dul@kmutt.ac.th; 3 Department of Chemical Engineering, Faculty of Engineering, King Mongkut’s University of Technology Thonburi, Bangkok 10140, Thailand; kunrapat60@gmail.com; 4 Division of Biotechnology, School of Bioresources and Technology, King Mongkut’s University of Technology Thonburi, Bangkok 10150, Thailand; supapon.che@kmutt.ac.th; 5 Biochemical Engineering and Pilot Plant Research and Development (BEC) Unit, National Center for Genetic Engineering and Biotechnology, National Science and Technology Development Agency, King Mongkut’s University of Technology Thonburi, Bangkok 10150, Thailand

**Keywords:** *Arthrospira platensis* C1, bioethanol, cyanobacteria, genome-scale metabolic model, glycogen

## Abstract

Glycogen-enriched biomass of *Arthrospira* *platensis* has increasingly gained attention as a source for bioethanol production. To study the metabolic capabilities of glycogen production in *A. platensis* C1, a genome-scale metabolic model (GEM) could be a useful tool for predicting cellular behavior and suggesting strategies for glycogen overproduction. New experimentally validated GEM of *A. platensis* C1 namely *i*AK888, which has improved metabolic coverage and functionality was employed in this research. The *i*AK888 is a fully functional compartmentalized GEM consisting of 888 genes, 1,096 reactions, and 994 metabolites. This model was demonstrated to reasonably predict growth and glycogen fluxes under different growth conditions. In addition, *i*AK888 was further employed to predict the effect of deficiencies of NO_3_^−^, PO_4_^3−^, or SO_4_^2−^ on the growth and glycogen production in *A. platensis* C1. The simulation results showed that these nutrient limitations led to a decrease in growth flux and an increase in glycogen flux. The experiment of *A. platensis* C1 confirmed the enhancement of glycogen fluxes after the cells being transferred from normal Zarrouk’s medium to either NO_3_^−^, PO_4_^3−^, or SO_4_^2−^-free Zarrouk’s media. Therefore, *i*AK888 could be served as a predictive model for glycogen overproduction and a valuable multidisciplinary tool for further studies of this important academic and industrial organism.

## 1. Introduction

Due to the environmental concerns of production and utilization of fossil fuels, researches towards renewable energy are currently of great interest. Conversion of carbohydrate-enriched biomass of microalgae has become one of the promising approaches for sustainable clean energy generation [1]. Since their carbohydrates are in the form of lignin-free cellulose, starch, or glycogen, microalgae are much easier to convert to monosaccharides compared to lignocellulosic feedstocks [2]. In particular, prokaryotic microalgae, cyanobacteria, have certain advantages over eukaryotic microalgae for many reasons. They possess peptidoglycan cell wall which is easily degraded by fermentation processes [3,4]. Moreover, cyanobacteria accumulate glycogen as storage carbohydrate which is an excellent feedstock for fermentation over starch [4]. Furthermore, transformation systems in cyanobacteria have been much better developed [5,6,7].

*Arthrospira* (*Spirulina*) *platensis*, a filamentous non-nitrogen-fixing cyanobacterium, is an attractive candidate which has certain properties to be used as promising feedstocks for bioethanol production. For example, it has the capacity to accumulate large amounts of glycogen during cultivation under nutrient or environmental stresses [1]. The highest glycogen productivity of 0.29 g L^−1^ d^−1^ was reported under NO_3_^−^ depletion and high light intensity of 700 μmol photons/m^2^/s [8]. It also has the unique impressive characteristics for industrial applications, including a contaminant-free culture under the outdoor cultivation [9] and a fast bio-flocculation capability under nutrient starvation condition [10]. Moreover, Aikawa et al. proposed a low cost technology that generates the highest yield of bioethanol from carbohydrate-rich *Arthrospira* biomass by yeast fermentation [3]. Although research works have showed the potential use of *Arthrospira* to generate bioethanol, however, the glycogen productivity is still not high enough to profitably produce bioethanol at the commercial scale. To overcome this challenge, strategies for enhancement of glycogen content in *A. platensis* is needed. Among *Arthrospira*, *A. platensis* strain C1 is a good candidate for bioethanol production because it has distinct advantages over the others in term of strain improvement, non-gliding property [9], genome sequence [11], transformation systems [12], and the most comprehensive data at the molecular level, transcriptomics [13,14] proteomics [15,16,17] and protein–protein interactions [18]. However, the glycogen production in *A. platensis* C1 has not been investigated.

Driven by advancements in high-throughput biological and computational technologies, genome-scale metabolic model (GEM), a mathematical form of the cellular metabolic network, is currently being the indispensable tool for understanding cell phenotypes and providing rational strategies to maximize production of a desired metabolic product [19]. GEMs of various organisms across three domains of life, i.e., archaea, bacteria, and eukarya have been constructed and applied in various research areas, ranging from industrial to medical biotechnology [20,21]. Flux balance analysis (FBA) [22] is the most commonly used approach to simulate the GEMs. To investigate the metabolic activity and integrate omics data for allowing comprehensive studies at the systematic level of *A. platensis* C1, the first GEM of *A. platensis* C1, *i*AK692, was developed in 2012 [23]. This model was applied to predict optimal growth behavior, metabolic phenotypes, and essential genes under autotrophic, heterotrophic, and mixotrophic growth conditions.

Since bioinformatics tools, pathway/genome databases, and literatures have now been updated, newly curated genes and biochemical knowledge have become available. In this research, an updated GEM of *A. platensis* C1 named *i*AK888 was employed. *i*AK888 was built based on information of *i*AK692 as well as new annotated and curated genomic and biochemical knowledge. The *i*AK888, a fully compartmentalized GEM, is the most up-to-date comprehensive model for *A. platensis*. The model was demonstrated herein to be a helpful tool for proposing the rational strategies for improvement of glycogen production in *A. platensis* C1.

## 2. Materials and Methods

### 2.1. Genome-Scale Metabolic Network Reconstruction

The *i*AK888 was reconstructed by refining and updating the previously reconstructed genome-scale model for *A. platensis* C1, *i*AK692 [23]. The first step in the metabolic network reconstruction process was genome reannotation, which provided the initial set of gene–protein reaction (GPR) associations. The draft genome sequence of *A. platensis* C1 (6.089 Mb; GenBank NZ_CM001632) [11] was retrieved. A functional annotation was then performed using two independent systems: (i) The rapid annotation of microbial genomes using Subsystems Technology (RAST) annotation servers [24] supported by the SEED [25] and (ii) Kyoto Encyclopedia of Genes and Genomes (KEGG) Automatic Annotation Server (KAAS) [26]. To increase confidence in the annotation, the predicted ORFs obtained from *i*AK692, RAST [18], and KAAS [26] were compared. Only the shared ORFs were considered and any conflicting annotations were discarded. All transport genes and reactions were identified using a BLAST search [27] against the Transporter Classification Database (TCDB) [28]. 

Subsequently, a list of GPR associations was assembled and manually curated to reflect actual physiology of *A. platensis* C1 based on various information sources including biochemical databases [25,29,30], literatures [31,32,33,34], and curated genome-scale models, *Synechocystis* sp. PCC6803 [35] and *Escherichia coli* [36]. Manual curation was performed according to the standard reconstruction protocol [37]. A confidence scoring system [37] was employed to assign a confidence score to every network reaction. The score reflects the amount of available evidence type, biochemical data, genetic data, physiological data, sequence data, and modeling data, associated with individual reaction, ranging from 1 to 4, where 1 is the lowest and 4 is the highest evidence score. The gene expression data of *A. platensis* C1 (accession E-MTAB-2714) [14] were acquired from ArrayExpress in order to verify existence of predicted ORFs involved in the reconstructed network. Briefly, gene expression data of the control sample, in which *A. platensis* C1 was cultured in Zarrouk’s medium at 35 °C under a light intensity of 100 µmol photons/m^2^/s, were preprocessed and normalized. Then, the average of the log intensities between a housekeeping gene, 16S rRNA, and all genes were compared. Genes whose expression were equal to or greater than the housekeeping gene indicated the evidence for the existence of these genes and the associated reactions. Furthermore, all reactions were charge and mass balanced. The reaction directions were also assigned based on the standard Gibbs free energy of reaction (Δ*_r_G*^o^) provided by the SEED [25]. Formulas and charges for all metabolites were checked against KEGG [29] and PubChem [38]. Subsequently, the cofactor specificity of the enzyme in *A. platensis* C1 was verified using organism-related species literature. Cellular compartments of candidate genes and reactions were determined based on the literature and the GEM of *Synechocystis* sp. PCC6803 [35]. Moreover, BLAST search [27] and functional domain analysis using Pfam [39] were conducted to refine missing and low confidence gene annotations. The ambiguous genes existing in the *i*AK692, but absent in the annotation results of RAST [24] and KAAS [26] were removed.

Additionally, energy-generating cycles (type-II pathways) or internal cycles (type-III pathways) were checked during the reconstruction process to ensure that ATP and NAD(P) could not be produced without nutrient consumption. For ATP, the ATP maintenance flux was optimized when CO_2_ and photon uptake fluxes were set to zero. For NAD(P), an artificial reaction NAD(P)H → NAD(P) + H was added in the reconstructed network and optimized when CO_2_ and photon uptakes were not available. If either ATP or NAD(P) could be produced without nutrient uptake, the reactions related to the production of these energy metabolite were checked manually.

An organism-specific biomass equation, representing cell growth in silico, was formulated and used as an objective function for simulating growth phenotypes through FBA [22]. The major macromolecular constituents of biomass synthesis consisted of protein, carbohydrate, lipid, DNA, RNA, pigments, vitamins, and minerals. The content of each constituent of *i*AK888, compared to the previously published model, *i*AK692, is shown in Table 1. The protein, carbohydrate, and lipid were estimated in the exponential growth phase of *A. platensis* C1 [40]. The stoichiometric coefficients of DNA, RNA, and protein synthesis reactions were estimated from the nucleotide and amino acid contents of the published genome of *A. platensis* C1 [11]. Moreover, the biomass equation for *i*AK888 also included pigments, vitamins and minerals, of which the coefficients were calculated based on the published information of the closest species [41,42,43,44,45]. In this study, all stoichiometric coefficients in the biomass equation were assumed to be constant under different environmental conditions. The detailed calculation of the biomass equation is provided in Supplementary File S1.

### 2.2. Flux Balance Analysis

Flux balance analysis (FBA) is a widely used constraint-based optimization approach for predicting specific reaction rates (fluxes) of a large-scale network based on stoichiometry and steady-state assumption [22]. Briefly, all reactions within a reconstructed genome-scale metabolic network were converted into a stoichiometric matrix, *S*, in which each column and row represented one unique reaction and metabolite, respectively. The entries of *S* are the stoichiometric coefficients of metabolites participating in a reaction. By applying the mass balance constraints and steady state assumption, the particular metabolite’s concentration change per unit time is equal to zero (*Sv* = 0), where *v* represents the vector of reaction fluxes. Then, other constraints such as thermodynamic and enzyme capacity were accounted, thereby, determining reaction directions (reaction directionality and reaction reversibility) [46]. Reaction directionality is typically assigned based on a negative Δ*_r_G*^o^ while, reaction reversibility is a kinetic property of enzymes which are able to catalyze the reactions in the forward and backward directions. Hence, thermodynamic constraints help to decide the reaction directions in FBA. Finally, a linear programming was applied to maximize or minimize an objective function, usually the biomass reaction flux. This optimization results in optimal objective flux and optimal flux distribution of the metabolic network. In this work, the COBRA toolbox version 2 [47] with MATLAB (The MathWorks, version R2015b) was employed to model and predict cell behaviors i.e., specific growth rate and glycogen production flux.

### 2.3. Estimation of Glycogen Production Flux

To represent the glycogen metabolism in *A. platensis*, *i*AK888 incorporates 4 glycogen associated reactions, i.e., (i) glycogen synthesis reaction, (ii) glycogen utilization reaction for biomass growth, (iii) glycogen transport reaction for carbon storage and (iv) glycogen degradation reaction. In this work, glycogen was treated as a monomer (C_6_H_10_O_5_, *M*_W_ = 162.141) in the glycogen synthesis reaction where one mole of glycogen was synthesized from one mole of glucose-1-phosphate by glycogen synthase (glgA, EC 2.4.1.21). In addition, glycogen phosphorylase (glgP, EC 2.4.1.1) that is responsible for the glycogen degradation was assumed to be inactive under autotrophic growth. Thus, the glycogen production flux was simply determined from the flux of the glycogen synthesis reaction.

### 2.4. Model Validation

To evaluate the accuracy of the reconstructed metabolic model, comparisons between predicted phenotypes and experimental data, including a maximal growth rate under different growth conditions and maximal carbohydrate production flux under nitrogen depletion condition were performed. 

For prediction of the maximum specific growth rate, the experimental data sets in which *A. platensis* C1 was grown under autotroph, heterotroph, and mixotroph were calculated and used as the input parameters. Cells grown under autotrophic conditions were simulated and compared, including two independent sets of *A. platensis* C1 experiments, (i) setting the photon uptake rate to 100 μmol photons/m^2^/s and HCO_3_^−^ uptake rate to 0.2 mmol/gDCW/h [23] and (ii) setting the photon uptake rate to 200 μmol photons/m^2^/s and HCO_3_^−^ uptake rate to 0.25 mmol/gDCW/h [48]. Cells grown under a heterotrophic condition were simulated by setting the photon uptake rate to zero and glucose uptake rate to 0.017 mmol/gDCW/h [23]. For mixotrophic condition, cell growth was simulated by setting the photon uptake rate to 100 μmol photons/m^2^/s, HCO_3_^−^ uptake rate to 0.2 mmol/gDCW/h, and glucose uptake rate to 0.017 mmol/gDCW/h [23].

To further assess the reliability of the model, the maximum total carbohydrate production flux under nitrogen depletion condition was predicted. The photon and HCO_3_^−^ uptake rate were set at 100 μmol photons/m^2^/s and 0.2 mmol/gDCW/h, respectively. NO_3_^−^ uptake rate and specific growth rate were set to zero as observed in the experiment [49]. The objective function was accounted for maximizing the carbohydrate production flux.

For all the simulations, the uptake rates of all nutrients present in the Zarrouk’s medium [50] were constrained as 0 to −1000, while the transport fluxes of CO_2_, O_2_, and H_2_O were left unconstrained. In addition, all photons were assumed to be absorbed and used for driving photosynthesis without the influence of photoinhibition.

### 2.5. Simulation of Glycogen Production Under Nutrient-Limited Conditions

The effect of NO_3_^−^, PO_4_^3−^, and SO_4_^2−^ on growth and glycogen production were simulated by FBA [22] under the autotrophic condition. The specific uptake rate of HCO_3_^−^ was fixed at 1.6 mmol/gDCW/h for all simulations to guarantee an excess carbon condition. The maximum photon uptake flux was set to 100 μmol photons/m^2^/s. The effect of each nutrient on specific growth rate and total glycogen production flux was analyzed by varying the uptake flux values of each nutrient, NO_3_^−^, PO_4_^3−^, and SO_4_^2−^. The objective function was set to maximize the flux of the glycogen synthesis reaction. Additionally, flux variability analysis (FVA) [51] was performed to determine the minimum and maximum possible fluxes under the simulation conditions. Geometric FBA [52] was also used to determine a unique optimal solution which is central to the range of possible flux distributions.

### 2.6. Experimental Validation 

To evaluate the validity of *i*AK888, *A. platensis* C1 (PCC9438) was cultured in 1 L Erlenmeyer flasks containing 500 mL of Zarrouk’s medium [50] at 35 °C under white fluorescent illumination at 100 μmol photons/m^2^/s until mid-logarithmic phase. Then, the cells were transferred to three different Zarrouk’s media, lacking either nitrate (NaNO_3_ and Co(NO_3_)·6H_2_O), or phosphate (K_2_HPO_4_), or sulfur (K_2_SO_4_, FeSO_4_·7H_2_O, MgSO_4_·7H_2_O, ZnSO_4_·7H_2_O, CuSO_4_·5H_2_O, NiSO_4_·7H_2_O, Ti(SO_4_), and K_2_Cr_2_(SO_4_)_4_·24H_2_O), and then re-incubated under the same incubation conditions as mentioned above. The control experiments were carried out in normal Zarrouk’s medium formulation [50]. Samples were collected at each time point 0, 3, 6, 12, 18, 24, 48, 72 and 96 hours after the cells were transferred to different media, and were kept frozen at −80 °C for further analysis. All experiments were repeated in triplicate.

To analyze growth of *A. platensis* C1, the cell concentration was quantified by turbidity based on the optical density at 560 nm (OD_560_) using a Genesys 20 spectrophotometer (Thermo scientific, Waltham, MA) and measured for dry cell weight (DCW). The correlation between OD_560_ and DCW (DCW in g/L = 1.0888xOD_560_) was calculated based on triplicated experiments. Glycogen content was measured using iodine-glycogen assay [53]. Briefly, 100 µL of wet cell was mixed with 50 mg of glass beads and 500 µL of phosphate-buffered saline. The samples were vortexed at maximum speed for 5 min and incubated at 65 °C for 10 min. The samples were then centrifuged at 12,000 rpm for 10 min at 4 °C. Subsequently, 100 µL of supernatants were mixed with 5 µL of iodine solution in Greiner 96-well plate and incubated at 25 °C for 1 min. Absorbance was determined using Microplate reader (Tecan Infinite M200, Mannedorf, Switzerland) at a wavelength of 492 nm. The glycogen concentration of the samples was calculated using the equation obtained from the linear regression of the standard curve. 

## 3. Results and Discussion

### 3.1. Reconstruction of the Updated Genome-Scale Metabolic Network of A. platensis C1

Genome annotation showed that the rapid annotation of microbial genomes using Subsystems Technology (RAST) annotation servers [24] predicted a total of 2626 (1759 unique genes), whereas the KEGG Annotation Server (KAAS) [26] predicted a total of 1729 genes (1011 unique genes). Using the previously published genome-scale reconstruction for *A. platensis* C1 (*i*AK692) [23] as a reference, the annotation results overlapping among these three sources were analyzed. There were 513 conserved genes while 70 genes were shared between *i*AK692 [23] and KAAS [26]; 45 genes were shared between *i*AK692 [23] and RAST [24]; 275 genes were shared between RAST [24] and KAAS [26] (Figure S1). These large overlaps found between sources indicated good annotation quality. Genes found in all sources or two of the sources were considered to have the high reliability. Regarding to the transport gene annotation, approximately 116 new transport genes in the genome encoding for transporters or transport-related proteins were obtained from KAAS [26] and TCDB [28]. This is because the transportation mechanisms in *i*AK692 [23] were only diffusion reactions. Thus, in this reconstruction, ABC transport reaction and symport or antiport reactions were addressed based on annotation-based inference of transporter function to represent the transport machinery of *A. platensis* C1. Subsequently, the GPR of all candidate genes were then manually curated based upon various information such as physiological evidence in literature, gene expression [14], and biochemical databases. Importantly, the reactions involved in glycogen biosynthesis and degradation pathways were elaborated. 

The reactions were balanced for charge and mass to prevent infeasible cycles. However, there were still mass- and charge-unbalanced reactions because either the associated metabolites contained an unspecified metabolite, R groups, or the correct reaction mechanism was unknown. During the curation, a metabolic gap was identified and filled through repeated cell growth simulations using FBA [22] until a positive flux on the biomass reaction was observed. These processes led to the modification of genes, reactions, and metabolites in *i*AK692 [23] and addition of new metabolic genes and their associated reactions in the updated model. For genes, 60 (6%) genes were removed, 268 (28%) genes were added, and 620 (66%) were refined. For reactions, 202 (15%) reactions were removed, 423 (33%) were added, and 673 (52%) were refined. For metabolites, 140 (12%) metabolites were removed, 297 (26%) were added, and 697 (62%) were refined (see Supplementary File S2 for details). Obviously, significant improvements in annotation resulted from not only re-annotation information collected from RAST [24] and KAAS [26] but also a manual effort to assess the reliability of such annotation. This is a crucial prior step to address the careful revision of the first genome-scale network reconstruction and derived constraint-based model of *A. platensis* C1 published in 2012 [23]. 

### 3.2. Characteristics of iAK888 and Comparison

**Figure 1:**
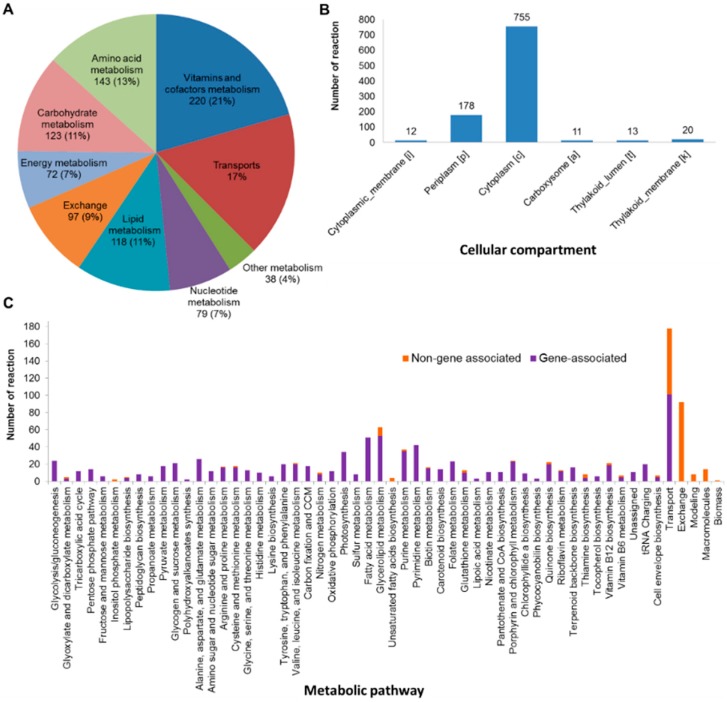
Properties of the updated genome-scale metabolic network of *A. platensis* C1. Distribution of reactions in each metabolism (**A**), Distribution of reactions in each cellular compartment (**B**), Gene associated and non-gene associated reaction in each metabolic pathway (**C**).

*i*AK888 contains 1096 metabolic reactions, 994 metabolites, and 888 genes-representing 15% of total protein coding genes in the genome [11]. Of all reactions, 751 (68.5%) were gene associated enzymatic reactions whereas non-gene associated enzymatic reactions, transport reactions and exchange reactions were 66 (6%), 182 (16.6%) and 97 (8.9%), respectively (Table 2). The reactions revealed 9 major subsystems (Figure 1A) including 56 metabolic pathways, as defined by KEGG [29]. Vitamins and cofactors metabolism and transport represented the largest portions of the network. These likely represented *A. platensis* physiology, an excellent source of vitamins [9] and reflected the fact that approx. 6% of *A. platensis* C1 genome encoded for transporters [11]. The reactions distributed over six cellular compartments including carboxysome, thylakoid lumen, thylakoid membrane, cytoplasmic membrane, cytoplasm and periplasm, with the majority of reactions localized to the cytosol (Figure 1B). This observation agreed well with the known life cycle of *A. platensis* [9]. Figure 1C showed the non-gene associated and gene associated reaction involved in each pathway. Notably, these non-gene associated reactions were required to complete the metabolic network of *A. platensis* C1 and were also observed in other GEMs [54,55]. Besides, the metabolites localized in different compartments of the fully compartmentalized model are considered as distinct metabolites. Therefore, without considering subcellular sites, the model accounted for 796 unique metabolites. Additionally, the quality of model reconstruction was assessed using the confidence scores associated to each reaction (Figure S2). The overall confidence score was 3.38. Almost 89% of the internal reactions (936) have been either very well or well-studied where extensive physiological and sequence evidences are available, while 11% were primarily based on the genome annotation and modeling hypotheses. The new metabolic network reconstruction for *i*AK888 is provided in a spreadsheet format (Supplementary File S3) that includes curation notes and references.

**Figure 2:**
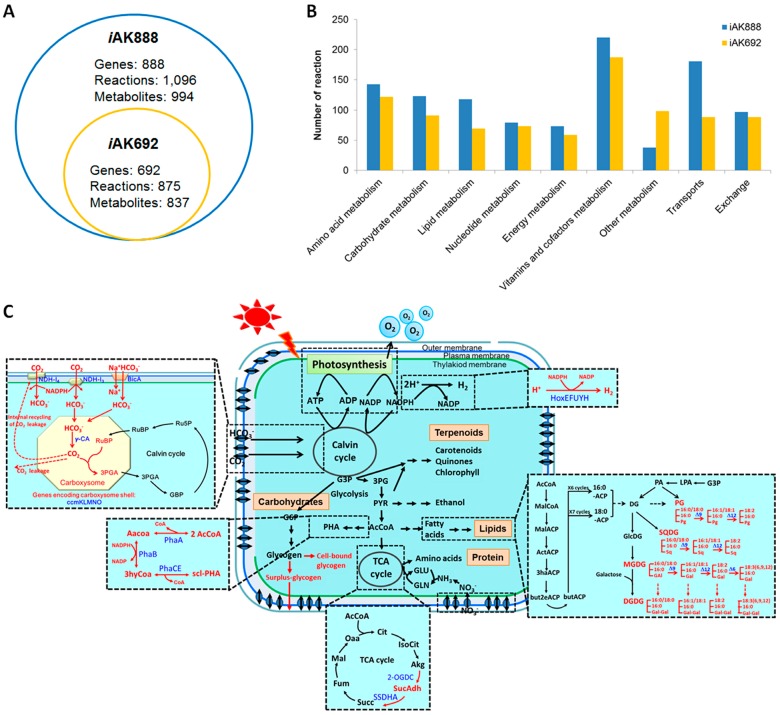
Comparison between *i*AK888 and *i*AK692. Overview features of *i*AK888 compared to *i*AK692 (**A**). Number of reactions in each metabolism of *i*AK888 compared to *i*AK692 (**B**). Schematic representation of *i*AK888 and the example of the filled pathways compared to *i*AK692 (**C**). Zoomed out sections are pathways that were completed in *i*AK888. Red texts and arrows indicate missing pathways in *i*AK692.

The properties of *i*AK888 were compared with the properties of two published GEM of *A. platensis* NIE-39 [55] and the first GEM of *A. platensis* C1 [23]. The *i*AK888 appeared to contain the largest number of genes, reactions, metabolites, and sub-cellular compartments (Table 2). It was evidently clear that the model reported in this research was the largest in terms of gene coverage. A comparison between *i*AK888 and *i*AK692 [23] specifically showed that *i*AK888 represented an increase in number of genes, reactions, and metabolites over *i*AK692 [23], by 196, 221, and 157, respectively (Figure 2A). Of the increased genes, 75 (38%) genes were the updated metabolic genes whereas 121 (62%) genes were the additional transport genes. The reactions associated to these genes covered a wide array of key metabolic functions mainly relevant to carbohydrate metabolism, lipid metabolism, vitamins and cofactors metabolism, and transports (Figure 2B). Furthermore, this effort was made to reconstruct some of the pathways which were either incomplete or not considered in the previous reconstruction such as tricarboxylic acid cycle (TCA) cycle, photosynthesis and oxidative phosphorylation, carbon concentrating mechanism (CCM), fatty acid biosynthesis, glycogen metabolism, polyhydroxyalkanoates biosynthesis, and hydrogen biosynthesis (Figure 2C). The incomplete TCA cycle in *i*AK692 [23] was improved based on the latest evidence reported in cyanobacteria [31,56]. Subsequently, the description of photosynthesis and oxidative phosphorylation was significantly improved according to a model organism, photosynthetic *Synechocystis* sp. PCC 6803 [35]. These included the photosynthetic linear electron flow (LEF) pathway [57], including photosystem I and II, alternate electron flow (AEF) pathways [58], and photorespiration [59]. In addition, the molecular components involved in CCM of *A. platensis* C1 were annotated and incorporated for a more precise understanding of the primary carbon metabolic route. There are 18 genes/proteins associated to the CCM in *i*AK888. Finally, reactions associated with polyhydroxyalkanoates and hydrogen biosynthesis were formulated to complement the physiological ability of *A. platensis* C1. In summary, *i*AK888 was considered to be the most comprehensive *A. platensis* model to date. 

### 3.3. Validation of iAK888

For the growth verification, results showed that the model accurately predicted the growth rates with the error less than 5% in all culture conditions (Table 3). Moreover, the model generated oxygen under autotrophic and mixotrophic growth conditions. The released oxygen content under autotroph was higher than mixotroph. On the other hand, the model consumed oxygen and produced CO_2_ under heterotrophic simulations. These results suggested that *i*AK888 was able to represent the basic behavior of *A. platensis* C1. The models in SBML format for all four growth conditions are provided in Supplementary File S4–7. Besides, flux distributions of these three growth conditions are presented in Supplementary File S8.

Furthermore, an effort was made to investigate the total carbohydrate production flux under NO_3_^−^ depletion. The simulation was performed to imitate the experimental conditions [49] with the following parameters: HCO_3_^−^ uptake rate, 0.2 mmol/gDCW/h; photon uptake rate, 100 μmol photons/m^2^/s; no growth and NO_3_^−^ uptake rate. The objective function was set to maximize the total carbohydrate production flux. Result showed that the *in silico* total carbohydrate production flux (0.0868 mmol/gDCW/h) was consistent with the experimental production (0.081 mmol/gDCW/h) [49]. The models in SBML format and flux distributions can be found in Supplementary File S9 and S10, respectively. These results suggested that *i*AK888 could predict the growth rates and carbohydrate production of *A. platensis* C1 reasonably well.

### 3.4. Prediction of Glycogen Overproduction Using iAK888

**Figure 3:**
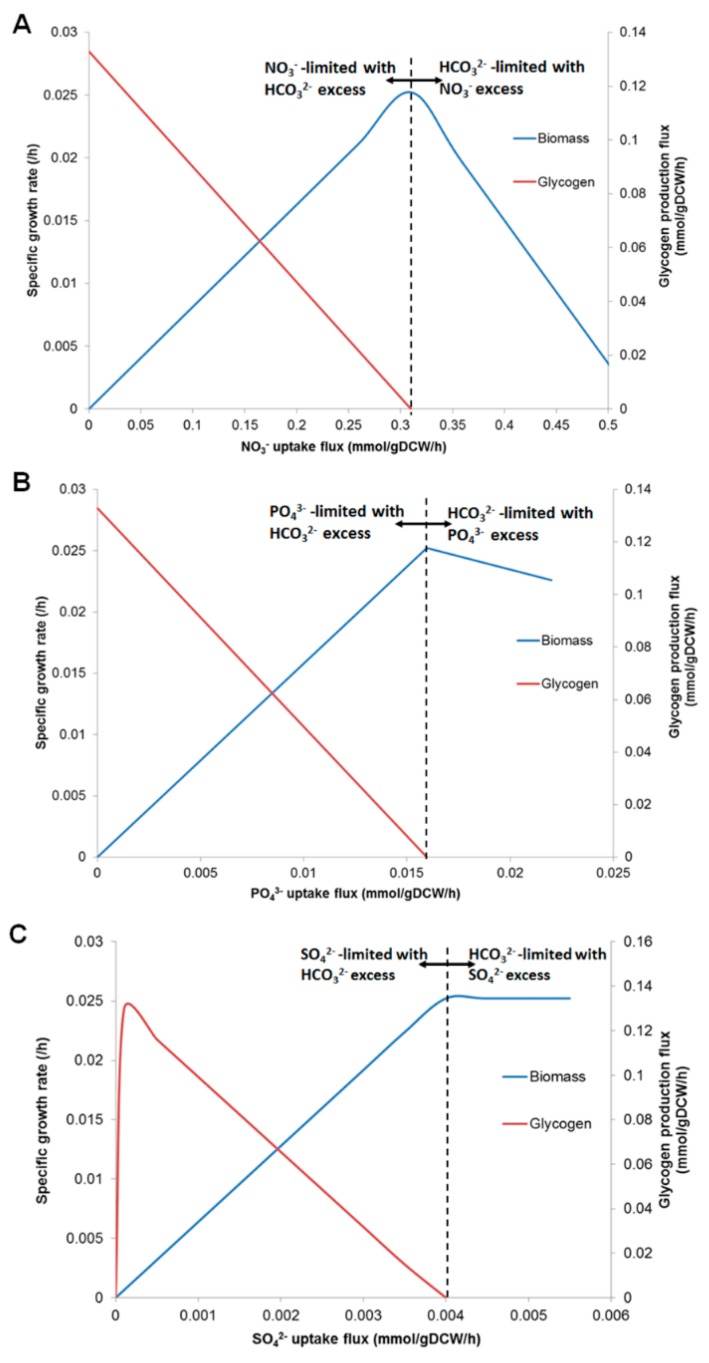
Prediction of nutrient effect on growth and glycogen production. The biomass and glycogen production as the functions of uptake fluxes of NO_3_^−^ (**A**), PO_4_^3−^ (**B**), and SO_4_^2−^ (**C**). Dashed line indicates the optimal uptake rate for growth.

Herein, the impacts of nutrient starvation on growth and glycogen production were simulated to predict cultivation strategies for enhancing glycogen content in *A. platensis* C1. *i*AK888 was simulated by varying the uptake flux of NO_3_^−^, PO_4_^3−^, and SO_4_^2−^ under autotrophic conditions with the following parameters: HCO_3_^−^ uptake flux, 1.6 mmol/gDCW/h; photon uptake flux, 100 μmol photons/m^2^/s. In overall, limitation of NO_3_^−^, PO_4_^3−^, and SO_4_^2−^ uptakes and the excess HCO_3_^−^ resulted in the negative effect on growth, while revealed positive effect on glycogen production (Figure 3A–C). Obviously, the specific growth rate rapidly decreased when limited levels of NO_3_^−^, PO_4_^3−^, and SO_4_^2−^ were introduced. Apparently, *i*AK888 expressed ability to rapidly accumulate glycogen when the uptake flux of each nutrient was lower than the optimal uptake flux for biomass production. On the other hand, when the uptake flux of each nutrient was higher than the optimal uptake flux for biomass production, the glycogen production flux exhibited no flux values.

**Figure 4:**
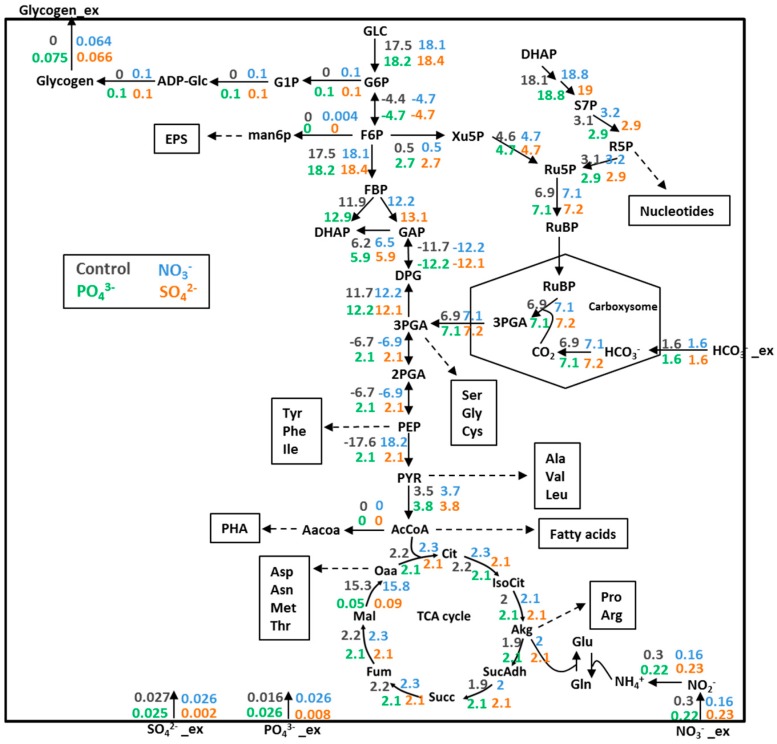
Illustrative fluxes in central metabolic predicted by geometric FBA for each growth condition: Control (black), NO_3_^−^-limited (NO_3_^−^, blue), PO_4_^3−^-limited (PO_4_^3−^, green) and SO_4_^2−^-limited (SO_4_^2−^, orange). The diagram included key reactions including the glycolysis, the carbon fixation through CO_2_-concentrating mechanism, the nonoxidative pentose phosphate pathway, the TCA cycle, and biosynthetic pathways of glycogen. Single and double-headed arrows indicate reactions assumed to be irreversible and reversible, respectively. Numbers labeled at the corresponding arrows show flux values in mmol/gDCW/h.

The internal metabolic fluxes throughout the central metabolism were determined using geometric FBA [52]. HCO_3_^−^ uptake rate was constrained to 1.6 mmol/gDCW/h whereas, NO_3_^−^, PO_4_^3−^, and SO_4_^2−^ were constrained to half of the optimum uptake rate for growth. Flux map (Figure 4) showed that all reactions in the central metabolism were activated to provide maximum biomass. The excess carbon was secreted as glycogen under NO_3_^−^, PO_4_^3−^, and SO_4_^2−^-insufficient growth. Fluxes comparison under different perturbations provided information on how central metabolic reactions respond to the altered growth condition, although each perturbation resulted in the similar overall phenotype. They exhibited different flux patterns. All fluxes in the central metabolisms were higher under NO_3_^−^-limited growth than the normal growth considered as NO_3_^−^-sufficient condition. All fluxes in the TCA cycle and some reactions of the glycolysis and non-oxidative pentose phosphate pathway were lower under PO_4_^3−^, and SO_4_^2−^-limited growth than the normal growth. A consequence of increased fluxes and metabolites in the central carbon pathway under NO_3_^−^ starvation led to the enhanced synthesis of glycogen in *i*AK888. This showed some agreements with recent metabolomic observations during the glycogen production phase in *A. platensis* NIE-39 cultured under nitrate-free (Society of Toxicology) SOT medium [60]. The time-course analysis of the primary metabolites content revealed a transient increase of glucose-1-phosphate, glucose-6-phosphate, fructose-6-phosphate, 2-ketoglutarate, succinate, and malate. Besides, simulation of each nutrient limitation with excess carbon showed secretion of pyruvate, acetate, and lactate (see Supplementary File S11–13). In agreement with the experimental results, it was found that *A. platensis* NIES-39 cultured under nitrate-limited condition produced pyruvate, acetate, and lactate in the culture medium [55]. Regarding to PO_4_^3−^, and SO_4_^2−^ starvation, the simulation results showed that the changes in flux patterns of both nutrients were very similar. Obviously, limitation of these nutrients greatly affected the fluxes through phosphoglycerate mutase, enolase, pyruvate kinase, and malate dehydrogenase reactions in comparison to the normal growth and NO_3_^−^-limited conditions. These much lower fluxes occurred at the branch points where the precursor metabolites were drained for the synthesis of amino acid. However, future inclusion of such experimental data could be expected to verify the prediction of flux distribution under each nutrient limitation.

### 3.5. Experimental Validation of *i*AK888 Prediction for Glycogen Overproduction

**Figure 5:**
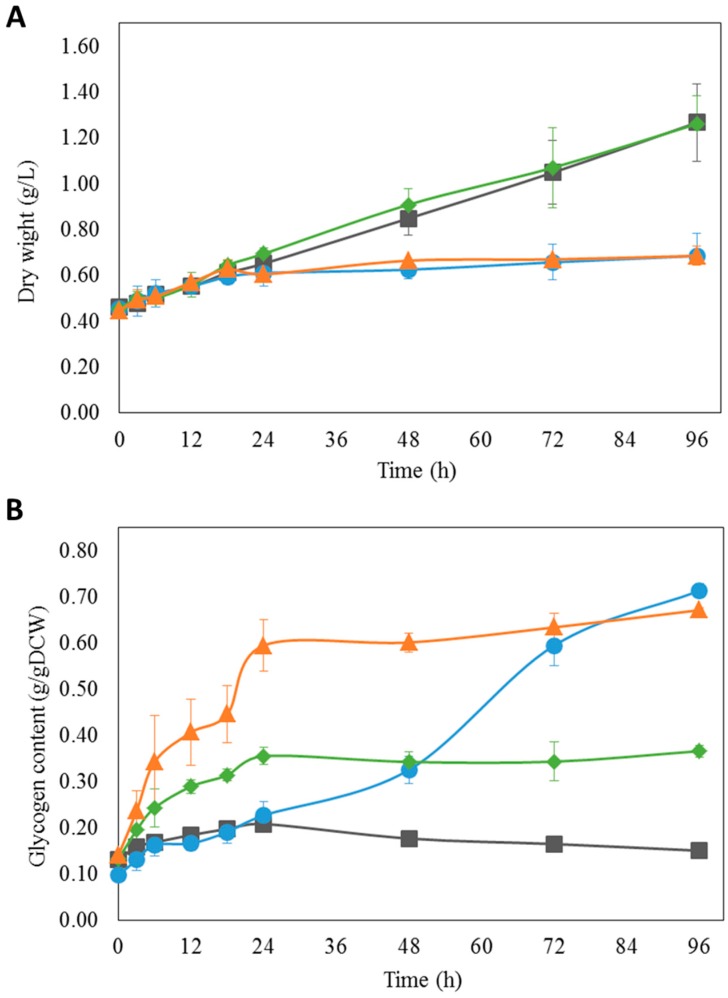
Growth (**A**) and glycogen content (**B**) of *A. platensis* C1 under nutrient starvation at 0–96 h. Culture conditions of Control (■), NO_3_^−^ (●), PO_4_^3−^ (♦) and SO_4_^2−^ (▲).

**Figure 6:**
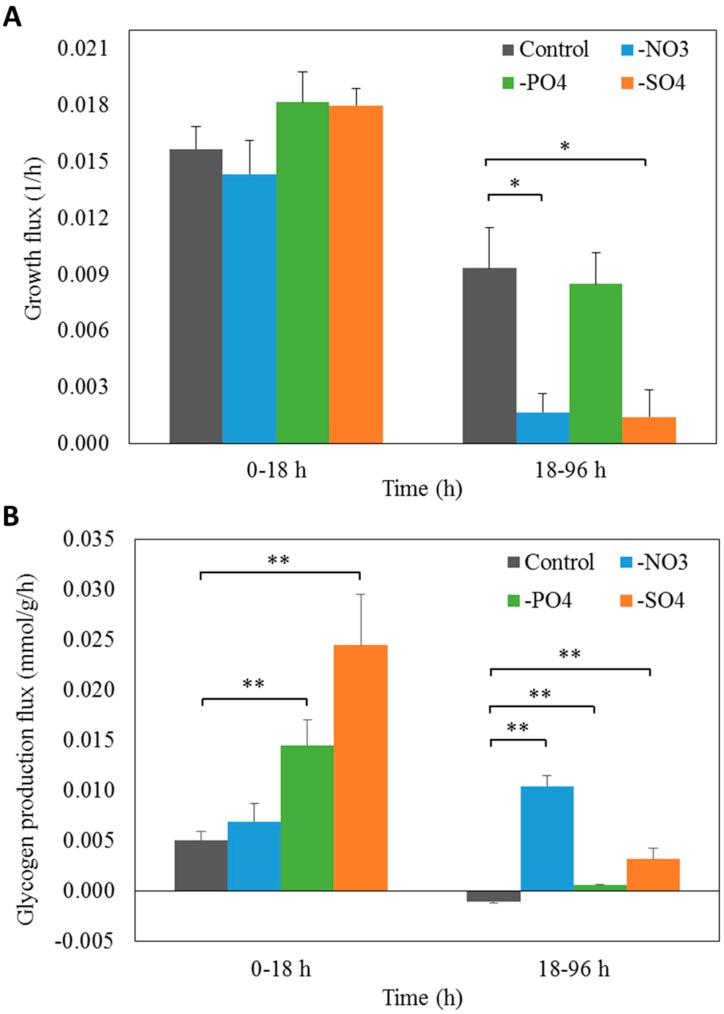
The specific growth rate growth (**A**) and the glycogen production flux (**B**) under the control, NO_3_^−^-depleted, PO_4_^3−^-depleted and SO_4_^2−^-depleted conditions. * and ** represent significant difference at the *p*-value < 0.05 and the *p*-value < 0.01, respectively.

Since *i*AK888 suggested that NO_3_^−^, PO_4_^3−^, and SO_4_^2−^ starvation enhance glycogen content in *A. platensis* C1, -growth rate and glycogen production under NO_3_^−^, PO_4_^3−^, and SO_4_^2−^ depletion was experimentally determined. *A. platensis* C1 was cultured with standard Zarrouk’s medium [50] and cells were transferred to nitrogen, phosphorus, or sulfur depleted Zarrouk’s medium [50]. Changes in cell growth and glycogen content of *A. platensis* C1 were investigated for 96 h. Regarding to growth in term of dry weight (Figure 5A) and growth rate (Figure 6A), the results showed no significant differences between control and experiments in the first 18 h. However, further incubation for 96 h, the dry weight and the growth rate under NO_3_^−^ and SO_4_^2−^ starvation decreased significantly (*p* < 0.05) whereas the dry weight under PO_4_^3−^ starvation gradually increased, even though the growth rate was lower than that of the control. An increase in growth observed during the first 18 h might be due to the results from availability of endogenous nitrogen, phosphorus, and sulfur. In terms of glycogen content (Figure 5B) and glycogen production flux (Figure 6B), cells subjected to PO_4_^3−^ and SO_4_^2−^ depletion showed a significant increase in glycogen content and production flux (*p* < 0.01) in the first 18 h. In contrast, the significant increase in glycogen content and production flux were observed in the NO_3_^−^-free medium after 24 h (*p* < 0.01).

Compared to the results predicted by FBA simulations, glycogen flux was considered to be consistent with those obtained by experiments, suggesting that NO_3_^−^, PO_4_^3−^, and SO_4_^2−^ depletion induced an increase of glycogen production in *A. platensis* C1. Notably, only the growth rate under PO_4_^3−^ starvation between the experimental and *i*AK888-simulated fluxes had no significant difference. It should be noted that despite FBA [22] being widely used approach for simulating a large-scale cellular metabolism under a certain condition, this method could only be used to predict the steady state snapshot of flux distribution. Thus, using the FBA approach [22] was unable to directly analyze the transient states of cell metabolism such as a concentration of metabolite and dynamic change in the flux with time. These reasons might cause the discrepancy between the FBA simulation and the experiment.

## 4. Conclusions

In this study, an improved genome-scale model for *A. platensis* C1, *i*AK888, was performed. The *i*AK888 model displayed a highly detailed reconstruction capturing the fundamental knowledge and the significantly biotechnological capabilities as compared to the previous model, *i*AK692. The *i*AK888 model was demonstrated to be a suitable model for prediction of growth and carbohydrate production flux of *A. platensis* C1 under various conditions. Moreover, it was demonstrated that the *i*AK888 model could suggest rational cultivation strategies for overproduction of glycogen in *A. platensis* C1. In the future, this new model shall be a versatile platform for further studies towards glycogen-enriched *A. platensis* C1 as an alternative feedstock for bioethanol production. 

## Figures and Tables

**Table 1:** Comparison of biomass constituents between *i*AK888 and *i*AK692.

Component	Content (%*w*/*w*)	
	*i*AK888	*i*AK692 [23]
Proteins	51.44	68
Carbohydrates	31.62	16
Lipids	4.98	11
DNA	0.88	0.88
RNA	3.12	3.12
Colorants	2.84	1
Vitamins	0.11	-
Minerals	2.79	-
Ash	2.27	-
Sum	100	100

**Table 2:** Comparison of network model characteristics of *A. platensis* species.

***Arthrospira* Species**	***A. platensis* C1**		***A. platensis* NIE-39**
**Genome Statistics**	-		-
Genome size (bp)	6,089,210		6,788,435
Protein coding genes	6108		6630
Gene with enzymes	952		905
Transporter genes	345		NA
**Model Name/Characteristics**	***i*AK888**	***i*AK692**	**-**
Total genes in model	888 (15%)	692 (11%)	620 (9%)
- Metabolic genes	767	692	579
- Transporter genes	121	0	41
Total biochemical reactions	1096	875	746
- Metabolic reactions	817	699	652
- Transport reactions	182	88	60
- Exchange reactions	97	88	34
Metabolites	994	837	673
Compartments	6	2	2
Reference	This study	[23]	[55]

**Table 3:** Comparison of in silico and experimental growth.

Growth Condition	Constraints of Consumed Metabolites			Maximal Specific Growth Rate (1/h)		% Error
	Photon Flux (μmol photons m^−2^ s^−1^)	HCO_3_^−^ Uptake Flux (mmol/g DCW/h)	Glucose Uptake Flux (mmol/g DCW/h)	Experiment	In Silico	
Autotroph	100	0.2	0	0.0255 [23]	0.0252	1.2
Autotroph	200	0.25	0	0.0331 [48]	0.0334	0.9
Heterotroph	0	0	0–0.017	0 [23]	0	0
Mixotroph	100	0.2	0–0.017	0.0262 [23]	0.0260	0.8

## Supplementary Materials

The following are available online at http://www.mdpi.com/2218-1989/8/4/84/s1. Figure S1: The Venn diagrams illustrate the overlapped genes among RAST [24], KAAS [26], and *i*AK692 [23]. Figure S2: Heat map of the confidence score of each pathway in *i*AK888. The columns represent the level of confidence score (ranging from 1 to 4) and the rows represent the metabolic pathway. The various colors correspond to the percentage of pathway reactions that have the corresponding confidence score (orange = 100%, blue = 0%). Based on the confidence score system [37], 4 represents physiological and genetic evidence, 3 represents direct and indirect evidence for gene function, 2 represents indirect evidence from physiological data or only sequence-based evidence, and 1 represents modeling evidence. Supplementary File S1: Biomass composition of *A. platensis* C1. Supplementary File S2: Genome annotation. Supplementary File S3: Detailed GPR information of model *i*AK888. Supplementary File S4: SBML of autotrophic growth photon uptake 100 μmol photons/m^2^/s. Supplementary File S5: SBML of autotrophic growth photon uptake 200 μmol photons/m^2^/s. Supplementary File S6: SBML of heterotrophic growth. Supplementary File S7: SBML of mixotrophic growth. Supplementary File S8: Flux distribution of autotrophic, heterotrophic, and mixotrophic growth. Supplementary File S9: SBML of carbohydrate production under NO_3_^−^ depletion. Supplementary File S10: Flux distribution of effect NO_3_^−^ depletion on carbohydrate production. Supplementary File S11: Flux distribution of effect NO_3_^−^ depletion on growth and glycogen. Supplementary File S12: Flux distribution of effect PO_4_^3−^ depletion on growth and glycogen. Supplementary File S13: Flux distribution of effect SO_4_^2−^ depletion on growth and glycogen.
