# Supplementary figures and images for: An Improved Genome-Scale Metabolic Model of Arthrospira platensis C1 (iAK888) and Its Application in Glycogen Overproduction
Source: Metabolites. 2018 Nov 26;8(4):84. doi: 10.3390/metabo8040084 (PMC6315860; doi:10.3390/metabo8040084)

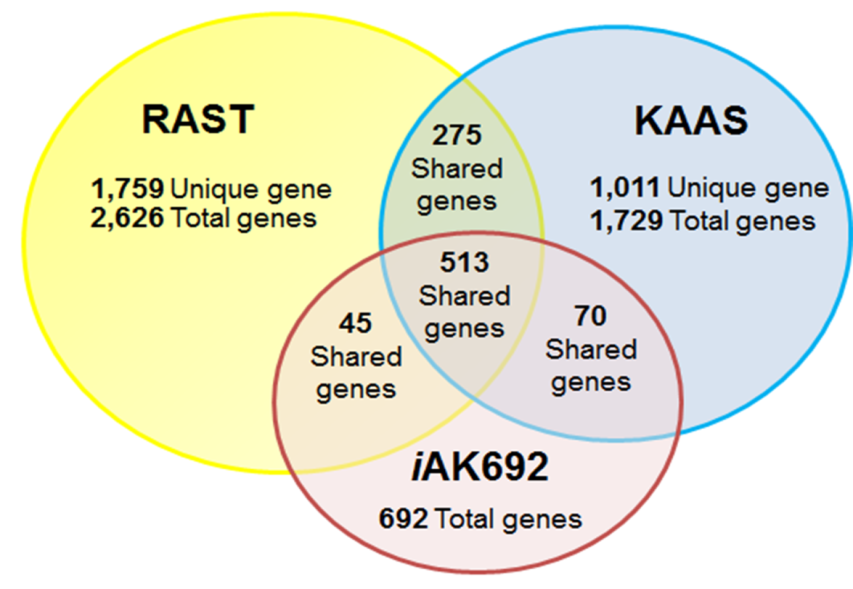

Supplement: Supplementary file 1 [file metabolites-08-00084-s001.zip › metabolites-384075-revised-suppl/Supplements/Figure S1.tif]

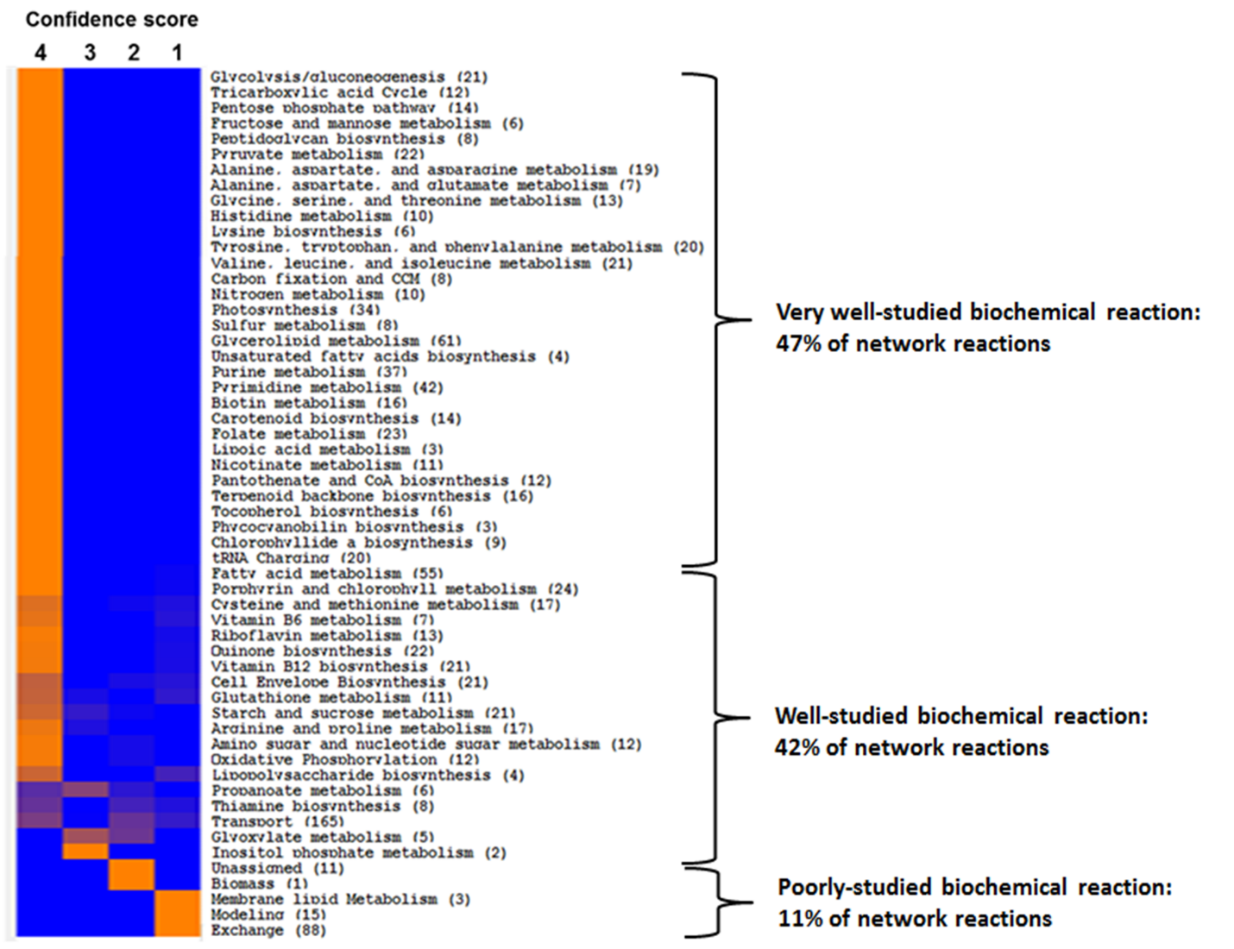

Supplement: Supplementary file 1 [file metabolites-08-00084-s001.zip › metabolites-384075-revised-suppl/Supplements/Figure S2.tif]
